# Transovarial transmission of *Babesia ovis* in *Rhipicephalus bursa*, confirmed by multi-generational experiments

**DOI:** 10.1051/parasite/2025055

**Published:** 2025-09-18

**Authors:** Sezayi Ozubek, Mehmet Can Ulucesme, Munir Aktas

**Affiliations:** Department of Parasitology, Faculty of Veterinary Medicine, University of Fırat Elazig 23200 Türkiye

**Keywords:** *Babesia ovis*, Experimental infection, Ovine babesiosis, *Rhipicephalus bursa*, Transovarial transmission

## Abstract

*Babesia ovis* is a tick-transmitted intraerythrocytic apicomplexan parasite that causes ovine babesiosis, a disease with high economic impact in endemic regions. Although *Rhipicephalus bursa* is a known biological vector of *B. ovis*, the dynamics of vertical (transovarial) transmission are poorly understood. In the present study, the transovarial transmission of *B. ovis* was investigated experimentally in four continuous generations of *R. bursa* under laboratory controlled conditions. A total of 11 sheep were used during the study. An initial stabilate co-infected with *B. ovis* and *Theileria ovis* was inoculated into a splenectomized sheep, followed by an infestation with *Babesia-*free adult *R. bursa*. Study of dead tick bodies and larval pools by molecular methodology confirmed the elimination of *T. ovis* and the vertical transmission *B. ovis*. *Rhipicephalus bursa* ticks derived vertically from F_1_ to F_4_ generations kept high levels of infection (98% in F_3_ adults), and consistently led to severe clinical babesiosis in both immunosuppressed and immunocompetent sheep. Although all feeding stages became infected while feeding on their hosts, only adult ticks were able to transmit the parasite to vertebrate hosts. Our findings demonstrate that *B. ovis* can be transmitted vertically through several tick generations, while preserving its ability to cause severe disease, even without selective pressure. The virulent, mono-infected *B. ovis* strain developed in this study will provide an infectious challenge model for future vaccine and pathogenesis studies under field-relevant conditions.

## Abstract (fr)

*Babesia ovi*s est un parasite apicomplexe intraérythrocytaire transmis par les tiques et responsable de la babésiose ovine, une maladie à fort impact économique dans les régions endémiques. Bien que *Rhipicephalus bursa* soit un vecteur biologique connu de *B. ovis*, la dynamique de la transmission verticale (transovarienne) est mal comprise. Dans la présente étude, la transmission transovarienne de *B. ovis* a été étudiée expérimentalement sur quatre générations continues de *R. bursa* dans des conditions contrôlées en laboratoire. Au total, 11 moutons ont été utilisés au cours de l’étude. Un stabilat initial co-infecté par *B. ovis* et *Theileria ovis* a été inoculé à un mouton splénectomisé, suivi d’une infestation par des adultes de *R. bursa* indemnes de *Babesia*. L’étude des tiques mortes et des lots de larves par méthodologie moléculaire a confirmé l’élimination de *T. ovis* et la transmission verticale de *B. ovis*. Les tiques *R. bursa* dérivées verticalement des générations F_1_ à F_4_ ont conservé des niveaux d’infection élevés (98 % chez les adultes F_3_) et ont systématiquement entraîné une babésiose clinique sévère chez les moutons immunodéprimés et immunocompétents. Bien que tous les stades d’alimentation aient été infectés en se nourrissant de leurs hôtes, seules les tiques adultes ont pu transmettre le parasite à leurs hôtes vertébrés. Nos résultats démontrent que *B. ovis* peut être transmis verticalement par plusieurs générations de tiques tout en préservant sa capacité à provoquer une maladie grave, même en l’absence de pression sélective. La souche virulente et mono-infectée de *B. ovis* développée dans cette étude fournira un modèle infectieux pour de futures études de vaccin et de pathogénèse dans des conditions de terrain.

## Introduction

*Babesia ovis* is an apicomplexan protozoan transmitted by ticks and is the etiological agent of ovine babesiosis, a disease characterized by anemia, high fever, reduced productivity, and, in severe cases, mortality in sheep [22, 26]. This disease has a significant economic burden in endemic regions, particularly in parts of southern Europe, the Middle East, and Asia [2, 9, 17, 25]. The principal vector of *B. ovis* is *Rhipicephalus bursa* Canestrini & Fanzago, 1878, a widely distributed ixodid tick species with a strong ecological association with small ruminants [28, 29]. In this life cycle, larvae and nymphs typically feed on the same host, while transmission of *B. ovis* only occurs through the adult tick stage [19].

Although the vector competence of *R. bursa* for *B. ovis* has been well documented [5, 6, 10, 11, 20], the mechanisms underlying parasite persistence in tick populations, particularly the possibility of vertical (transovarial) transmission, remain inadequately clarified. In contrast to *Theileria* spp., which undergo only transstadial development within the vector, *Babesia* spp. are capable of both transstadial and transovarial transmission [21, 22]. Nonetheless, direct experimental confirmation of transovarial transmission of *B. ovis* under controlled conditions is limited. Previous reports suggesting the transovarial transmission of *B. ovis* by *R. bursa* are historically limited and often lack molecular confirmation using modern diagnostic tools [8, 15].

To address this gap, we aimed to experimentally investigate whether *B. ovis* can be vertically transmitted across multiple generations of *R. bursa* under controlled laboratory conditions. In addition, since the initial infection material contained both *B. ovis* and *Theileria ovis*, we aimed to evaluate whether vertical passage would result in the elimination of *T. ovis*, which is not known to be vertically transmitted. This would also enable the establishment of a pure *B. ovis*-infected tick line, which may serve as a useful tool for downstream studies on transmission, pathogenesis, and vaccine development.

## Materials and methods

### Experimental design and animal use

This study was conducted to demonstrate the transovarial transmission of *B. ovis* across four generations of *R. bursa*. The sterile *R. bursa* ticks used in this study were obtained from a laboratory-maintained colony established in our laboratory and originally sourced from Elazig, Türkiye [27]. Briefly, species identification was performed under a stereomicroscope using standard taxonomic keys. The colony was maintained under sterile conditions, and all generations were routinely screened for *B. ovis* and other tick-borne pathogens. Specifically, nPCR was performed for *Babesia*, *Theileria*, *Anaplasma*, and *Ehrlichia* species using specific primers, as previously described [26]. Only ticks that tested negative for all pathogens were used in experimental infestations. All procedures involving animals were approved by the Local Ethics Committee for Animal Experiments of Firat University, Animal Experiment Ethic Committee, under protocol number 2023/12-05.

A total of 11 sheep aged 5–8 months and four New Zealand rabbits were used throughout the experimental timeline. Splenectomy was performed under general anesthesia using aseptic surgical procedures at least three weeks before tick infestation to allow full recovery and stabilization of hematological parameters [24]. The immunosuppressed sheep received intramuscular dexamethasone (Vetakort^®^ 4 mg/mL, Vetas, Istanbul, Türkiye) at a daily dose of 20 mg for ten consecutive days prior to infestation to promote susceptibility to infection.

All animals were housed individually in tick-proof isolation units under veterinary supervision. Feed and water were provided *ad libitum*, and animals were monitored daily for general health and clinical signs throughout the study.

### Stabilate origin and molecular characterization

The initial *B. ovis* stabilate used in this study was obtained from a naturally infected 2-year-old female sheep presenting with clinical signs of babesiosis, including anemia, jaundice, and hemoglobinuria. The case originated from the Alacakaya district of Elazig province in eastern Türkiye. Giemsa-stained peripheral blood smears prepared from the ear tip revealed intraerythrocytic *Babesia* parasites, with an estimated parasitemia (PPE) of 1%. Approximately 50 mL of blood was collected into tubes containing ethylenediaminetetraacetic acid (EDTA) and mixed with 10% dimethyl sulfoxide (DMSO) as a cryoprotectant. The mixture was aliquoted into cryovials and preserved in liquid nitrogen for later use as a stabilate.

To identify the causative piroplasm species, DNA was extracted from 200 μL of infected blood using a PureLink™ Genomic DNA Mini Kit (Invitrogen, Thermo Fisher Scientific, Waltham, MA, USA). Reverse Line Blot (RLB) hybridization assay targeting the V4 hypervariable region of the *18S rRNA* gene was performed according to the method described by Ozubek & Aktas (2017) [18], and revealed a mixed infection with *B. ovis* and *T. ovis*. Since *T. ovis* cannot be transmitted transovarially, a tick passage approach was employed to eliminate co-infection and obtain a *B. ovis* stabilate.

### Tick acquisition and transovarial initiation (F_0_ Generation)

**Figure 1:**
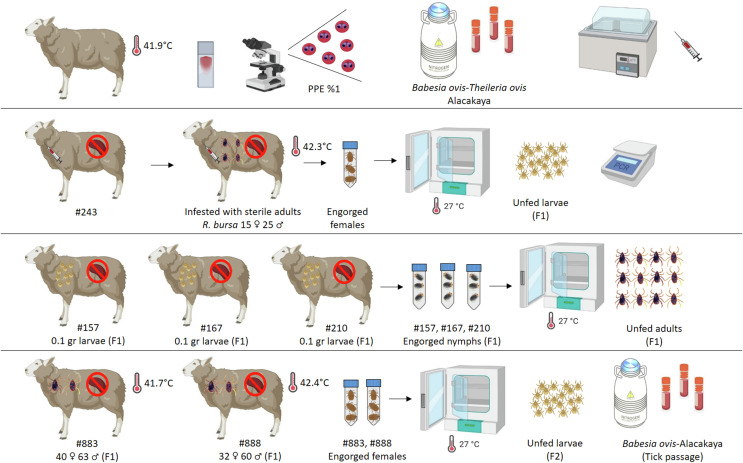
Schematic representation of the first two generations (F_0_–F_1_) in the experimental transovarial transmission of *B. ovis* by *R. bursa*. The process led to the development of F_2_ larvae infected only with *B. ovis*, and the generation of virulent *B. ovis* (Alacakaya) stabilate.

A splenectomized sheep (#243) was intravenously inoculated with 15 mL of the cryopreserved mixed (*B. ovis*-*T. ovis*) stabilate. A single feeding capsule was attached to the chest region of the animal on the day following experimental inoculation, and 40 *Babesia*-free, unfed adult *R. bursa* ticks (15 females and 25 males), were introduced into the capsule [4, 27] to synchronize tick feeding with the anticipated rise in parasitemia. Daily clinical monitoring was performed. Rectal temperature and parasitemia (PPE) (based on Giemsa-stained blood smears) were recorded. Ticks completed feeding and engorged females were collected on day 9 post-inoculation and incubated at 27 ± 1 °C and 70–80% relative humidity to allow oviposition. After egg-laying, bodies of female ticks were bisected longitudinally and stored at –20 °C for DNA extraction. Four larval pools (~100 larvae per pool) were generated from each female. DNA was extracted from both female bodies and larval pools and analyzed by RLB. Larvae showing vigorous motility and confirmed to be *B. ovis*-positive by nPCR were selected. Approximately 0.1 g of larvae from each selected pool were applied via capsule to three splenectomized sheep (#157, #167, and #210) as described by Almazán *et al.* (2018) [4]. After completion of larval feeding, engorged nymphs were collected individually from each sheep and incubated under standard conditions to obtain unfed adult ticks (F_1_ generation) (Fig. 1).

### First transovarial tick transmission and pathogenicity testing (F_1_ generation)

Adult *R. bursa* ticks of the F_1_ generation were placed on two splenectomized sheep (#883 and #888) in individual feeding capsules. A total of 103 unfed adults (40 females and 63 males) was applied on sheep #883 and 92 unfed adults (32 females and 60 males) on sheep #888. All the animals of both groups were observed on a daily basis for clinical and parasitic status. The parameters assessed were rectal temperature, mucosal membrane, hematocrit (HCT) levels, and PPE estimated by microscopic examination of Giemsa-stained blood smears.

Blood samples were also obtained periodically for molecular diagnosis by nPCR and RLB assay to detect *B. ovis* DNA (see Molecular Methods section for details). Engorged female ticks that had finished feeding were collected from each of the sheep, transferred to the laboratory and maintained at 27 ± 1 °C with 70–80% relative humidity, where they oviposited. The larvae produced were considered the F_2_ generation and utilized for the subsequent experiment (Fig. 1).

### Second transovarial generation and confirmation of vertical passage (F_2_ generation)

The F_2_ generation of larvae from engorged F_1_ females was fed on a New Zealand rabbit (Rabbit #1) by a feeding capsule system. Engorged nymphs were collected from Rabbit #1 and held in controlled conditions (27±1 °C, 70–80% RH) until they molted into adults. These F_2_ adults were used to infest three sheep (two splenectomized: #588, #676, and one immunosuppressed intact: #473). An amount of 20 mg of intramuscular dexamethasone (Vetakort^®^ 4 mg/mL, Vetas) was administered to sheep #473 once a day for 10 days post-tick infestation to suppress the immune system. All sheep were examined on a daily basis for clinical and parasitic status via rectal temperature, HCT level, and PPE by microscopy. At various times during the infection process, *B. ovis* infection was confirmed using nPCR and RLB. Engorged female ticks were collected and incubated under standard conditions for oviposition to generate the F_3_ larval generation.

### Third transovarial generation and molecular verification (F_3_ generation)

**Figure 2:**
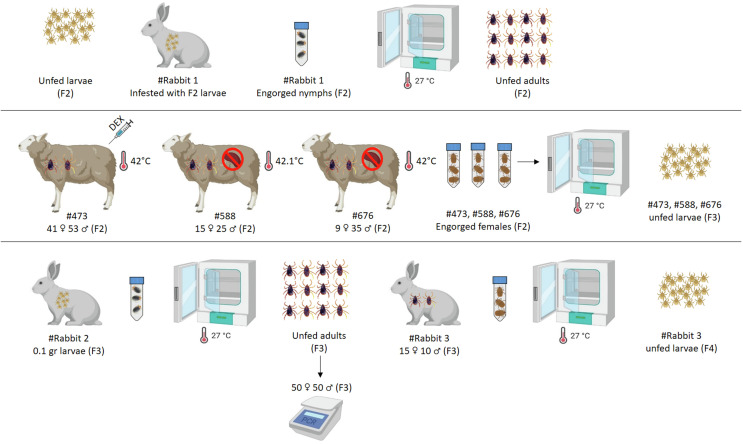
Experimental progression from F_2_ to F_4_ generation in the transovarial transmission of *B. ovis* by *R. bursa*. F_2_ larvae were cycled through rabbit and sheep hosts to produce infected adult ticks. Molecular screening of 100 F_3_ adults confirmed *B. ovis* infection and the absence of *T. ovis*. Resulting F_4_ larvae were successfully generated for subsequent transmission experiments.

**Figure 3:**
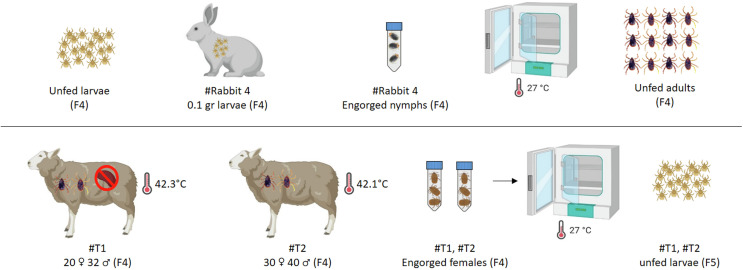
The final stage of the transovarial transmission experiment, showing the development of F_4_ tick and transmission to sheep. F_4_ larvae were cycled through Rabbit #4 and applied to a splenectomized sheep (#T1) and an immunocompetent sheep (#T2). Both hosts supported tick feeding and the production of F_5_ larvae, confirming sustained transovarial transmission of *B. ovis* through four generations.

F_3_ larvae, obtained from F_2_ females that had oviposited, were applied to a New Zealand rabbit (Rabbit #2) using a capsule-based feeding system. After blood feeding, engorged nymphs were collected and incubated at 27 ± 1 °C with 70–80% relative humidity until they molted into adult ticks. A randomly selected subset of 100 F_3_ adult ticks (50 males, 50 females) was individually tested using the nPCR and RLB hybridization assay to assess infection status. Subsequently, F_3_ adults were applied to Rabbit #3 to continue the tick life cycle. Fully engorged females were collected after feeding, incubated under standard conditions, and allowed to oviposit. The resulting F_4_ larvae were applied to Rabbit #4 for blood feeding, after which engorged nymphs were collected and incubated to obtain unfed F_4_ adults (Figs. 2 and 3).

### Fourth transovarial generation and final transmission to sheep (F_4_ generation)

Unfed F_4_ adult ticks were applied on individual feeding capsules to two sheep: one splenectomized (#T1) and one immunocompetent (#T2). Tick infestation and attachment were recorded and animals were observed for 25 days post-infestation. Clinical examinations were performed daily and consisted of rectal temperature, examination of mucous membranes, HCT, and detection of parasitemia by microscopic examination of Giemsa-stained peripheral blood smears. Blood samples were taken at various time points for molecular detection of *B. ovis* using nPCR and RLB.

### Genomic DNA extraction, PCR, and data analysis

Genomic DNA extractions from both blood and tick tissues were performed using a PureLink™ Genomic DNA Mini Kit (Invitrogen), strictly adhering to the manufacturer’s protocol. For blood-derived samples, 200 μL of whole blood were processed to ensure consistent and high-purity DNA recovery. In the case of ticks, adult female specimens were dissected longitudinally following oviposition, and one half of each carcass was individually placed into Eppendorf tubes. These samples were cryogenically ground in liquid nitrogen to facilitate complete homogenization and subsequently stored at –20 °C until DNA extraction [3].

The isolated DNA was then used as a template in nPCR assays targeting *Babesia*, *Theileria*, *Anaplasma*, and *Ehrlichia* spp., as well as species-specific detection of *B. ovis*, using established primer sets and protocols cited in previous studies [1, 7, 12, 14, 16]. A full list of primer sequences used in this study, along with their target genes, expected amplicon sizes, and references, is provided in Supplementary Table 1. Initial PCR reactions (25 μL) included standard components such as 10× PCR buffer (VitaTaq, Procomcure Biotech, Thalgau, Austria), dNTPs, Taq polymerase, target-specific outer primers (Nbab1F-Nbab1R; Ec9-Ec12a), and genomic DNA. In the nested step, 1 μL of the first-round product was amplified under identical reagent conditions. Each reaction series incorporated appropriate positive controls (*B. ovis* – EF092454.1, *A. ovis* – MG642087.1) and a negative control (nuclease-free water) to validate the assay. Amplified DNA fragments were separated by electrophoresis on 1.4% agarose gels using TAE buffer and visualized with a Quantum Gel Imaging System (Vilber Lourmat, Marne-la-Vallée, France). Samples testing positive for *Babesia*/*Theileria* or *Anaplasma*/*Ehrlichia* DNA by nPCR were subsequently subjected to RLB analysis to confirm species identity. For RLB, 20 μL of each positive PCR product was diluted to 150 μL in 2× SSPE/0.1% SDS buffer, denatured at 95 °C for 10 min, cooled on ice, and hybridized to genus- and species-specific probes immobilized on an RLB membrane, as previously described [17, 18].

Data visualizations, including time-course curves and scatter distributions, were prepared in GraphPad Prism version 8 (GraphPad Software, San Diego, CA, USA), while schematic figures (Figs. 1–3) illustrating experimental workflows were created using https://www.biorender.com/.

## Results

### Confirmation of initial infection and tick acquisition (F_0_ Generation)

**Figure 4:**
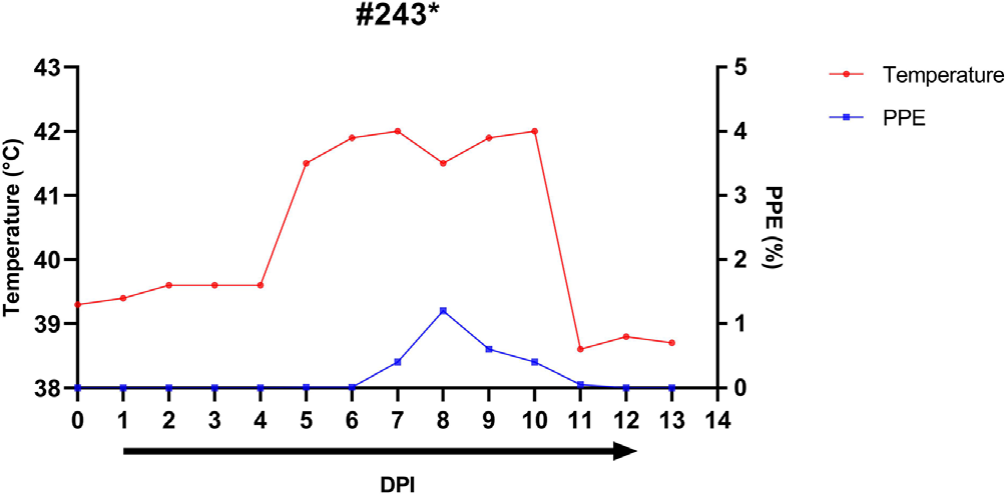
Rectal temperature (°C) and PPE (%) profile of sheep #243 over the course of infection. The black horizontal arrow indicates the period during which adult *R. bursa* ticks were allowed to feed for pathogen acquisition (days 1–12 post-inoculation) (*: splenectomized).

This study successfully demonstrated the transovarial transmission of *B. ovis* through four consecutive generations of *R. bursa* under controlled experimental conditions. After sheep #243 was inoculated, the clinical and parasitological follow-up showed a response typical of an acute *B. ovis* infection. The rectal temperature was recorded and remained in the physiological range (39.3–39.6 °C) in the first 4 days post-inoculation; there was a significant febrile response on day 5 (41.5 °C), and it reached 42 °C on days 7 and 10. Parasitemia first became microscopically visible on day 5, increasing rapidly to 1.2% on day 8, and then decreasing. This PPE interval coincided with the period of active tick attachment feeding (days 4–12), which is the most likely period for optimal acquisition of *B. ovis* by adult *R. bursa* ticks (Fig. 4). RLB analysis of engorged female bodies confirmed co-infection with *B. ovis* and *T. ovis*, whereas larval pools were positive only for *B. ovis*, indicating vertical acquisition of *B. ovis* and the absence of transovarial transmission of *T. ovis* at the F_0_ generation. For the F_1_ generation, only fully engorged adult ticks were tested by nPCR and RLB; partially fed ticks were not included in the molecular screening.

**Figure 5:**
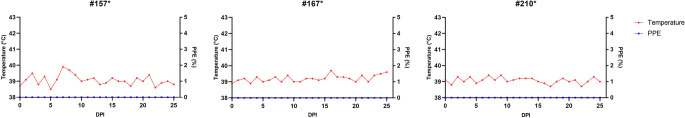
Rectal temperature (°C) and PPE (%) profiles of splenectomized sheep (#157, #167, and #210) following infestation with F_0_ generation *R. bursa* larvae (*: splenectomized).

### Pathogenicity and transmission of F_1_ generation ticks

Selected F_0_ larvae were used to infest splenectomized sheep (#157, #167, and #210). None of the animals exhibited clinical signs including fever, anemia, and hemoglobinuria in the course of observation for 25 days. Furthermore, blood samples collected from the animals were repeatedly tested by microscopy (Giemsa-stained blood films), nested PCR (nPCR), and RLB, and all results remained negative throughout the follow-up period.

**Figure 6:**
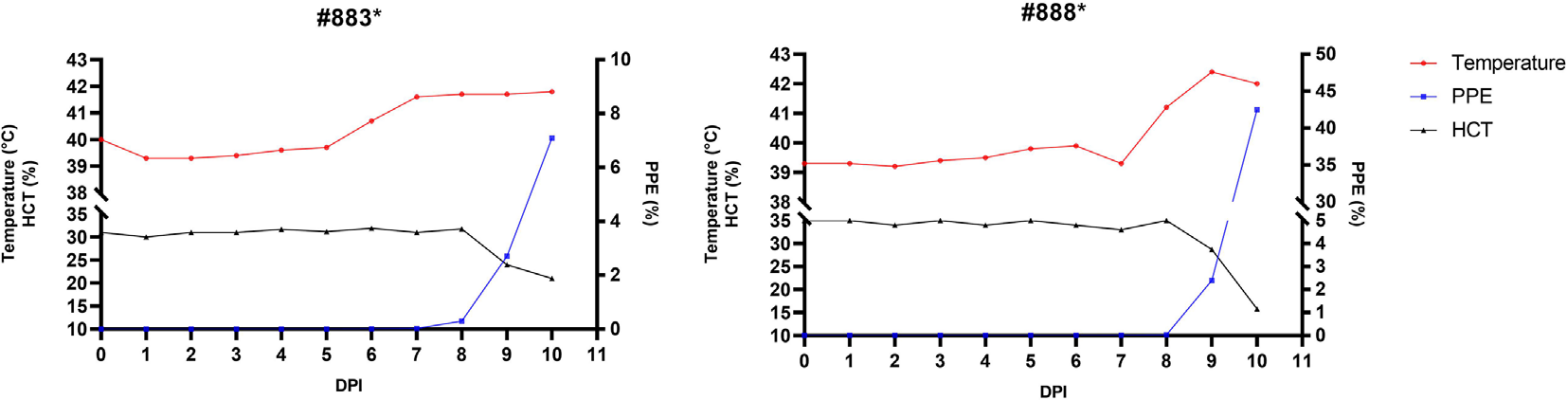
Clinical and hematological progression of *B. ovis* infection in splenectomized sheep #883 and #888 following infestation with F_1_ adult *R. bursa* ticks. Monitored parameters included rectal temperature (red), PPE (blue), and HCT (black) over a 10-day period (*: splenectomized).

Following larval feeding and nymphal development, F_1_ adult ticks were obtained and subsequently applied to splenectomized sheep #883 and #888. Parasitemia was detected in both animals within one week post-infestation. In sheep #883, rectal temperature began to increase on day 6 up to 40.7 °C and peaked on day 10 at 41.8 °C. Detection of PPE began on day 6 and rapidly increased to 7.1% on day 10. HCT values decreased to 31.9% on day 6, and to 21% on day 10, indicative of severe anemia. In sheep #888, a moderate fever (41.2 °C) developed on day 8 and peaked at 42.4 °C on day 9. The PPE pattern was comparable and appeared on day 7; after which there was a rapid rise to 42.5% by day 10. There was a similar decrease in HCT levels, which fell from 35% on day 5 to 15.8% on day 10 (Fig. 6). Confirmed *B. ovis* infection was recognized in both animals by molecular testing with nPCR and RLB. Both animals succumbed to infection on day 10 post infestation. Due to the acute course of infection, necropsy revealed only mild icteric changes, with no advanced systemic lesions observed.

### Transmission by F_2_ generation and clinical outcomes

**Figure 7:**
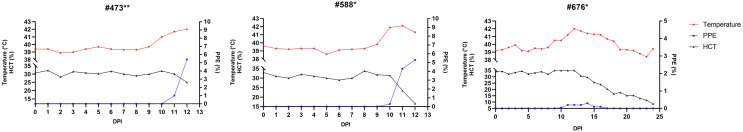
Clinical progression of *B. ovis* infection in sheep #473, #588, and #676 following infestation with F_2_ adult *R. bursa* ticks. Parameters monitored include rectal temperature (red), PPE (blue), and HCT (black) (*: splenectomized, **: immunosuppressed with dexamethasone).

All engorged female ticks from sheep #883 and #888 were collected on day 10 post-infestation and incubated under controlled conditions (27 ± 1 °C, 70–80% RH) to allow oviposition. The resulting larvae constituted the F_2_ generation. F_2_ larvae were cycled through Rabbit #1 and molted into adult ticks. These F_2_ adults were then applied to three sheep, two of which were splenectomized (#588 and #676) and one immunosuppressed (#473). All animals were monitored daily for temperature, PPE, and HCT. In sheep #473, PPE was first detected on day 10 post-infestation and peaked at 5.4% by day 12. This increase coincided with a sharp rise in rectal temperature (42 °C) and a decline in HCT from 32.2% to 25%, indicating acute babesiosis in the dexamethasone-treated animal. In sheep #588, PPE emerged on day 9 and increased to 5.3% by day 12. Fever reached 42.1 °C, and HCT dropped significantly from 33.8% to 16.6%, reflecting rapid onset hemolytic anemia and active infection. In contrast, sheep #676 exhibited a more prolonged but progressive course. Parasitemia appeared at day 9, peaked modestly at 0.3% by day 14, and gradually resolved. Despite lower parasite load, HCT values steadily declined from 35% to 8.6% by day 24, suggesting cumulative anemia from prolonged subclinical infection. All three animals succumbed to severe clinical babesiosis; #588 and #473 on day 12, and #676 on day 24 post-infestation (Fig. 7). Necropsy examinations revealed mild icterus, and marked systemic anemia, confirming the fatal outcome of *B. ovis* infection transmitted by F_2_ generation *R. bursa* ticks.

**Figure 8:**
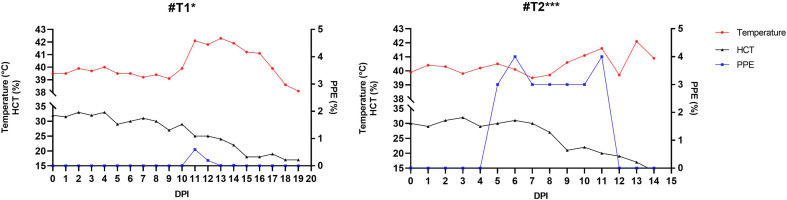
Clinical, parasitological, and hematological profiles of sheep #T1 and #T2 following infestation with F_4_ generation *R. bursa* ticks. Parameters include rectal temperature (red), HCT (black), and PPE (blue) over the post-infestation period (*: splenectomized, ***: spleen-intact).

### High infection prevalence in F_3_ generation ticks

Engorged female ticks collected from sheep #473, #588, and #676 produced F_3_ larvae, which were fed on Rabbit #2 using a capsule-based system. After engorgement and molting, F_3_ adult ticks were obtained. To verify vertical transmission in this generation, 100 individual F_3_ adults (50 females and 50 males) were tested by RLB hybridization. The analysis revealed 98% positivity for *B. ovis*, while *T. ovis* was not detected in any of the screened ticks, confirming stable and specific transovarial transmission. These verified F_3_ adults were subsequently fed on Rabbit #3 to generate F_4_ larvae. The resulting F_4_ larvae were then applied to Rabbit #4. After completion of the feeding and incubation period, unfed F_4_ adult ticks were successfully obtained.

### Transmission and clinical impact of F_4_ generation ticks

F_4_ adult ticks were infested by capsule on two sheep, one splenectomized (#T1) and one immunocompetent (#T2). Clinical babesiosis appeared in both animals 5–6 days after infestation. In sheep #T1, PPE was initially observed on day 10 of infection and reached a maximum of 0.6% on day 11. Parasitemia values fell thereafter but the animal had a high persistent fever (up to 42.3 °C on day 13; fever was defined as rectal temperature exceeding 40.0 °C) and HCT decreased over time (from 32% to 17%). The animal succumbed on the 19th day post-infestation. Sheep #T2 underwent a more rapid course. PPE stood at 4% on day 11 with rectal temperature reaching a maximum value of 42.1 °C, while HCT decreased from 30% to 14% during the 13-day period. The animal succumbed to infection on the 14th day. The infection of both animals with *B. ovis* was confirmed by microscopy and nPCR, and RLB. Pathological examination showed typical findings of acute babesiosis with marked splenomegaly, mild icterus, and anemia. These results demonstrate the retention of virulence and consistent vertical transmission of *B. ovis* for four successive generations of *R. bursa*. The overall infection status across generations, host species, and testing results is summarized in Table 1.

**Table 1:** Experimental animal records and tick generation data supporting transovarial transmission of *B. ovis*.

Animal *ID*	Splenectomized	*Dex	Max temp (°C)	Max PPE (%)	Prepatent period (days)	PPE duration (days)	Outcome	Tick generation	Tick count (♀/♂)
#243	Yes	No	42.0	1.2	5	6	Survived	F_0_	15/25
#157	Yes	No	39.9	-	-	0	No infection	F_1_ Larvae	0.1 g larvae
#167	Yes	No	39.7	-	-	0	No infection	F_1_ Larvae	0.1 g larvae
#210	Yes	No	39.4	-	-	0	No infection	F_1_ Larvae	0.1 g larvae
#883	Yes	No	41.8	7.1	6	5	Fatal	F_1_ Adult	40/63
#888	Yes	No	42.4	42.5	7	5	Fatal	F_1_ Adult	32/60
#588	Yes	No	42.1	5.3	9	4	Fatal	F_2_ Adult	15/25
#676	Yes	No	42.0	0.3	9	9	Fatal	F_2_ Adult	9/35
#473	No	Yes	41.7	5.4	10	3	Fatal	F_2_ Adult	41/53
#T1	Yes	No	42.3	0.6	10	10	Fatal	F_4_ Adult	20/32
#T2	No	No	42.1	4.0	5	7	Fatal	F_4_ Adult	30/40

* Dexamethasone.

## Discussion

This study presents the comprehensive experimental validation of sustained transovarial transmission and maintenance of *B. ovis* by *R. bursa* across four consecutive generations under controlled laboratory conditions. While transstadial transmission has been demonstrated experimentally in prior studies [10], our findings provide concurrent and longitudinal evidence for transovarial transmission modes. Vertically infected larvae successfully retained the parasite through their development into nymphs and adults and successive generations.

Although earlier works, such as Büscher *et al.* (1988) [8], demonstrated the presence of *B. ovis* in tick ovaries, hemolymph, and eggs through microscopy, they did not verify the infectivity or generational continuity of the pathogen in vertebrate hosts. Similarly, the long-term study by Markov and Abramov (1970) [15], which maintained *B. ovis* infection in *R. bursa* over 44 generations via selection of infected females, showed the potential for vertical persistence, but lacked assessment of clinical outcomes and molecular confirmation. On the other hand, our study documented vertical transmission over four complete tick generations (F_0_–F_4_) in the absence of laboratory-induced selection pressure. Molecular testing at every stage of the experiment using nPCR and RLB analysis indicated high stability of infection, with *B. ovis* detected in 98% in F3 adult ticks. Importantly, although the primary stabilate used to establish infection was co-infected with *T. ovis*, only *B. ovis* was successfully vertically passaged. The absence of *T. ovis* in subsequent tick generations resulted in the derivation of a pure and virulent *B. ovis* stabilate, providing an important resource for further transmission, pathogenesis, and vaccine studies.

Our results are consistent with previous findings that immature ticks infected with *B. ovis* were ineffective at transmitting *B. ovis* to intact and splenectomized sheep [11]. Our study identified that despite verified immature tick infection, they were unable to transmit the infection in splenectomized sheep. Only adult ticks were efficient vectors of *B. ovis* leading to clinical disease. This is in line with the notion that although immature ticks can be transient hosts of this pathogen, successful transmission to vertebrate hosts is predominantly accomplished by adult ticks.

Moreover, the sustained virulence of *B. ovis* across all four tick generations in our study highlights the epidemiological relevance of vertical transmission. Notably, lethal infections occurred in vertebrate hosts even in the absence of repeated exposure to infected blood meals, underscoring the capacity of vertically infected ticks to perpetuate transmission. While theoretical models and earlier studies have suggested that infection rates in tick populations associated solely with vertical transmission would decline over time, our findings challenge this assumption within the four-generation scope. In particular, previous work by Markov and Abramov (1970) [15] reported that although up to 95% of vertically infected female ticks produced infected eggs, fewer than 30% of those eggs were actually infected. In contrast, we observed a 98% infection rate among F_3_ adult ticks, indicating a markedly higher vertical transmission efficiency under our experimental conditions. This discrepancy may reflect differences in parasite strain, tick population genetics, or methodological factors, and collectively, our data support the robustness of *B. ovis* maintenance *via* vertical transmission under non-selective conditions.

In support of this, another recent study attempted to replicate natural infection conditions by experimentally infecting sheep with *B. ovis* and infesting them with *Babesia*-free *R. bursa* larvae six months later (representing the natural life cycle of *R. bursa*, which is one generation per year) [11]. This six-month interval corresponds to the time between peak clinical babesiosis and the larval questing period in the field. Under such conditions, however, larvae were unable to become infected from chronically infected hosts, supporting the idea that transstadial transmission alone was not enough to maintain *B. ovis* [11]. These results provide additional support for the biological requirement and epidemiological importance of transovarial transmission for the pathogen to be persistently maintained in endemic foci.

Interestingly, in our experimental model, the severity of anemia did not always correlate with the level of parasitemia. For example, some animals with low PPE (#676, PPE 0.3%) exhibited severe anemia and fatal outcomes, while others with higher PPE displayed similar or only slightly more severe clinical progression. This dissociation between PPE and hematological deterioration is consistent with the findings of Sevinc *et al.* (2013), who similarly reported no strong correlation between parasite load and the degree of anemia in naturally infected sheep [23]. Recent studies in human babesiosis models, including *Babesia divergens* infection, have demonstrated that *Babesia* not only invades red blood cells, but also induces a marked reduction in erythrocyte deformability, leading to impaired microcirculation, splenic clearance, and increased hemolysis [13]. These biomechanical alterations in infected RBCs may help explain why some animals experience rapid hematological decline despite low or moderate PPE.

Although different numbers of ticks were used across the animals in this study, the severity and onset of babesiosis did not appear to correlate directly with tick load. For instance, sheep #676 developed fatal babesiosis after exposure to a relatively small number of adult ticks (9♀/35♂), while other animals infested with more ticks showed a similar course of disease. This indicates that transmission and disease outcome may depend more on factors like tick infectivity, feeding success, or host susceptibility than on tick quantity alone. One important consideration in interpreting these results is the limited number of animals in each test group. While consistent trends were observed, increasing the number of animals would improve the reliability of the findings, help capture biological variation, and provide stronger support for the conclusions drawn. Expanding the sample size in future studies would allow a better understanding of how vertical transmission operates under different biological and ecological conditions.

Overall, our findings provide a comprehensive framework for understanding the role of transovarial transmission in the long-term maintenance of *B. ovis* by *R. bursa*. The combination of molecular proof, clinical surveillance, and generation-based tracing underlines the ability for ticks to become vertically infected and maintain virulent infection. While these results reinforce the biological and epidemiological relevance of vertical transmission, the study is limited to four generations under controlled laboratory conditions. Environmental variability, host heterogeneity, and the action of natural selection in natural ecosystems could potentially affect transmission success over longer temporal scales. Thus, field implementation studies taking into account genomic surveillance and ecological modeling are required to better evaluate the contribution of vertical transmission to disease persistence.

## Conclusion

This study experimentally verified transovarial transmission of *B. ovis* by *R. bursa* and showed the ability of the parasite to persist through several generations of its vector when acquired. By eliminating *T. ovis* co-infection and by consistently demonstrating pathogenicity over four generations, we derived genetically pure and virulent *B. ovis*-infected tick lines. These results provide compelling evidence for the involvement of vertical transmission in the persistence of *B. ovis* and emphasize the ability of *R. bursa* to serve as both a vector and a long-term reservoir under specific eco-epidemiological contexts. While laboratory conditions allowed for controlled and repeatable observation of vertical transmission, field-based research is necessary to determine how these patterns manifest under natural ecological pressures. Importantly, the mono-infected and virulent *B. ovis* line developed here represents a valuable challenge model for vaccine development, as it closely mimics field-relevant infection and disease patterns. Future investigations should aim to evaluate the dynamics of vertical transmission in endemic field settings, assess long-term genetic stability of *B. ovis* across multiple tick generations, and explore the use of the established mono-infected tick line in vaccine efficacy trials.

## Supplementary material

Supplementary Table 1: Primers and sequences used in this study for the detection of tick-borne pathogens. The supplementary material of this article is available at https://www.parasite-journal.org/10.1051/parasite/2025055/olm.
