# Supplementary material for: Transovarial transmission of Babesia ovis in Rhipicephalus bursa, confirmed by multi-generational experiments
Source: Parasite. 2025 Sep 18;32:60. doi: 10.1051/parasite/2025055 (PMC12445847; doi:10.1051/parasite/2025055)
Supplement: Supplementary file 1 — Supplementary Table 1: Primers and sequences used in this study for the detection of tick-borne pathogens. [file parasite-32-60-s1.pdf]

Supplementary Table 1: Primers and sequences used in this study for the detection of tick-borne pathogens

Tick-borne pathogens		Target gene	Primer	Primer sequence (5′–‘3)	Product size (bp)	Reference
Anaplasma / Ehrlichia	1st reaction	*16S rDNA*	Ec9<br>Ec12A	TACCTTGTTACGACTT<br>TGATCCTGGCTCAGAACGAACG	1462	[4]
	nPCR		16S8FE<br>BGA1B	GGAATTCAGAGTTGGATC(A/C)TGG(C/T)TCAG<br>CGGGATCCCGAGTTTGCCGGGACTT(C/T)TTCT	492-498	[2]
Babesia /Theileria	1st reaction	*18S rDNA*	Nbab1F<br>Nbab1R	AAGCCATGCATGTCTAAGTATAAGCTTTT<br>CTTCTCCTTCCTTTAAGTGATAAGGTTCAC	1600	[5]
	nPCR		RLBF2<br>RLBR2	GACACAGGGAGGTAGTGACAAG<br>CTAAGAATTTCACCTCTGACAGT	390-430	[3]
Babesia ovis	1st reaction	*18S rDNA*	Nbab1F<br>Nbab1R	AAGCCATGCATGTCTAAGTATAAGCTTTT<br>CTTCTCCTTCCTTTAAGTGATAAGGTTCAC	1600	[5]
	nPCR		BboF<br>BboR	TGGGCAGGACCTTGGTTCTTCT<br>CCGCGTAGCGCCGGCTAAATA	549	[1]

1. Aktaş M, Altay K, Dumanli N. 2005. Development of a polymerase chain reaction method for diagnosis of Babesia ovis infection in sheep and goats. Veterinary Parasitology, 133, 277–281.
2. Bekker CP, De Vos S, Taoufik A, Sparagano OA, Jongejan F. 2002. Simultaneous detection of Anaplasma and Ehrlichia species in ruminants and detection of Ehrlichia ruminantium in Amblyomma variegatum ticks by reverse line blot hybridization. Veterinary Microbiology, 89, 223–238.
3. Georges K, Loria GR, Riili S, Greco A, Caracappa S, Jongejan F, Sparagano O. 2001. Detection of haemoparasites in cattle by reverse line blot hybridisation with a note on the distribution of ticks in Sicily. Veterinary Parasitology, 99, 273–286.
4. Kawahara M, Rikihisa Y, Lin Q, Isogai E, Tahara K, Itagaki A, Hiramitsu Y, Tajima T. 2006. Novel Genetic Variants of *Anaplasma phagocytophilum* , *Anaplasma bovis* , *Anaplasma centrale* , and a Novel *Ehrlichia* sp. in Wild Deer and Ticks on Two Major Islands in Japan. Applied and Environmental Microbiology, 72, 1102–1109.
5. Oosthuizen MC, Zweygarth E, Collins NE, Troskie M, Penzhorn BL. 2008. Identification of a Novel *Babesia* sp. from a Sable Antelope (*Hippotragus niger* Harris, 1838). Journal of Clinical Microbiology, 46, 2247–2251.
